# MicroRNA-31-5p Exacerbates Lipopolysaccharide-Induced Acute Lung Injury via Inactivating Cab39/AMPK*α* Pathway

**DOI:** 10.1155/2020/8822361

**Published:** 2020-10-08

**Authors:** Wan-li Jiang, Kao-chang Zhao, Wen Yuan, Fang Zhou, Heng-ya Song, Gao-li Liu, Jie Huang, Jin-jing Zou, Bo Zhao, Song-ping Xie

**Affiliations:** ^1^Department of Thoracic Surgery, Renmin Hospital of Wuhan University, Wuhan 430060, China; ^2^Department of Pulmonary and Critical Care Medicine, Renmin Hospital of Wuhan University, Wuhan 430060, China; ^3^Department of Laboratory Medicine, Wuhan Medical and Health Center for Women and Children, Huazhong University of Science and Technology, Wuhan 430016, China; ^4^Department of Anesthesiology, Renmin Hospital of Wuhan University, Wuhan 430060, China

## Abstract

Acute lung injury (ALI) and the subsequent acute respiratory distress syndrome remain devastating diseases with high mortality rates and poor prognoses among patients in intensive care units. The present study is aimed at investigating the role and underlying mechanisms of microRNA-31-5p (*miR-31-5p*) on lipopolysaccharide- (LPS-) induced ALI. Mice were pretreated with *miR-31-5p* agomir, antagomir, and their negative controls at indicated doses for 3 consecutive days, and then they received a single intratracheal injection of LPS (5 mg/kg) for 12 h to induce ALI. MH-S murine alveolar macrophage cell lines were cultured to further verify the role of *miR-31-5p* in vitro. For AMP-activated protein kinase *α* (AMPK*α*) and calcium-binding protein 39 (Cab39) inhibition, compound C or lentiviral vectors were used in vivo and in vitro. We observed an upregulation of *miR-31-5p* in lung tissue upon LPS injection. *miR-31-5p* antagomir alleviated, while *miR-31-5p* agomir exacerbated LPS-induced inflammation, oxidative damage, and pulmonary dysfunction in vivo and in vitro. Mechanistically, *miR-31-5p* antagomir activated AMPK*α* to exert the protective effects that were abrogated by AMPK*α* inhibition. Further studies revealed that Cab39 was required for AMPK*α* activation and pulmonary protection by *miR-31-5p* antagomir. We provide the evidence that endogenous *miR-31-5p* is a key pathogenic factor for inflammation and oxidative damage during LPS-induced ALI, which is related to Cab39-dependent inhibition of AMPK*α*.

## 1. Introduction

Acute lung injury (ALI) and the subsequent acute respiratory distress syndrome are devastating diseases manifested as severe refractory hypoxemia and multiple organ failure, which cause high mortality rates and poor prognoses among patients in the intensive care units. Despite the advances in mechanical ventilations and symptomatic therapies, no specific and effective management strategies are available for ALI patients [1, 2]. Lipopolysaccharide (LPS) is a major component of the outer membranes in Gram-negative bacteria and functions as a key pathogenic factor to induce sepsis-related ALI. Upon LPS exposure, the downstream nuclear factor kappa-B (NF-*κ*B) is activated to trigger the synthesis of multiple inflammatory mediators, including interleukin-1*β* (IL-1*β*) and tumor necrosis factor-*α* (TNF-*α*). These cytokines in turn recruit leukocytes (e.g., neutrophils and macrophages) to infiltrate into lung tissue and further amplify sepsis-induced inflammation and lung injury [3, 4]. Nucleotide-binding domain-like receptor protein 3 (NLRP3) inflammasome acts as a molecular scaffold for the maturation of various procytokines that contributes to inflammatory injury during sepsis-related ALI [3, 4]. Besides, these leukocytes and excessive inflammation also promote an overproduction of reactive oxygen species (ROS) and elicit oxidative damage to pulmonary cells. In contrast, oxidative stress augments leukocyte chemotaxis and NLRP3 activation, thereby accelerating proinflammatory cytokine production and pulmonary injury [5]. To scavenge these free radicals, many antioxidant enzymes are synthesized under the control of a redox-sensitive transcription factor, named nuclear factor-erythroid 2 related factor 2 (NRF2) [6]. Therefore, inhibiting inflammation and oxidative stress may provide an effective method for the prevention and treatment of ALI.

AMP-activated protein kinase *α* (AMPK*α*) is a highly conserved serine/threonine protein kinase among eukaryotic organisms and has diverse beneficial functions on energy modulation, mitochondrial homeostasis, autophagic flux, fibrotic remodeling, and cell death [7–9]. Recent studies indicate that AMPK*α* also plays critical roles in the pathogenesis of sepsis-induced ALI via regulating inflammation and oxidative stress. Lv et al. found that AMPK*α* activation suppressed intrapulmonary inflammation and oxidative damage, thereby preventing LPS-induced ALI, while conversely, AMPK*α* inhibition exacerbated lung injury in mice [10, 11]. Taken together, these findings provide a basis for targeting AMPK*α* as the promising strategy to treat sepsis-related ALI.

MicroRNAs (miRs) are a class of evolutionarily conserved, single-stranded short noncoding RNAs that regulate gene expression at posttranscriptional levels through binding to the 3′-untranslated regions (UTR) of targeted messenger RNAs [12, 13]. A number of researches have proved the necessity of microRNAs in modulating sepsis-induced inflammation, oxidative stress, and ALI [14, 15]. *miR-31-5p* is well accepted as an oncogenic microRNA and participates in the proliferation, migration, invasion, and chemosensitivity of cancer cells [16, 17]. Yet, Kim et al. found that *miR-31-5p* was elevated in TNF-*α*-treated human endothelial cells and defined it as a NF-*κ*B-responsive microRNA with inflammation-modulating actions [18]. Results from Toyonaga et al. indicated that *miR-31-5p* was upregulated in inflammatory bowel disease and was associated with colonic epithelial cell integrity and function [19]. Besides, a recent study reported the role of *miR-31-5p* on ROS accumulation in hepatocellular carcinoma [16]. Based on these data, we supposed that *miR-31-5p* might be involved in the pathogenesis of LPS-induced ALI.

## 2. Materials and Methods

### 2.1. Reagents

LPS from *E. coli* 0111:B4 and compound C (CpC) were purchased from Sigma-Aldrich (St. Louis, MO, USA). The *miR-31-5p* agomir, antagomir, and their negative controls (AgNC for agomir and AntaNC for antagomir) were synthesized by GenePharma (Shanghai, China). The agomir sequence was 5′-AGGCAAGAUGCUGGCAUAGCUG-3′, while the antagomir sequence was 5′-CAGCUAUGCCAGCAUCUUGCCU-3′. Assay kits for detecting the levels of IL-1*β*, IL-6, IL-18, TNF-*α*, malondialdehyde (MDA), 3-nitrotyrosine (3-NT), and 4-hydroxynonenal (4-HNE) and assay kits for detecting the activities of lactate dehydrogenase (LDH), myeloperoxidase (MPO), caspase-1, superoxide dismutase (SOD), catalase (CAT), and glutathione peroxidase (Gpx) were obtained from Abcam (Cambridge, MA, USA). Dichloro-dihydro-fluorescein diacetate (DCFH-DA) was purchased from Beyotime (Shanghai, China). Short hairpin RNAs against calcium-binding protein 39 (Cab39, also known as MO25*α*; sh*Cab39*) or the scramble control (sh*Ctrl*) was obtained from Santa Cruz Biotechnology (Dallas, Texas, USA) and then packaged to the lentiviral vectors. A bicinchoninic acid (BCA) protein assay kit was purchased from Abcam (Cambridge, MA, USA). The primary antibodies against the following proteins were purchased from Abcam (Cambridge, MA, USA): apoptosis-associated speck-like protein (ASC), NLRP3, Lamin B1, NRF2, and Cab39. Anti-phospho NF-*κ*B P65 (p-P65), anti-total NF-*κ*B P65 (t-P65), anti-p-AMPK*α*, anti-t-AMPK*α*, and anti-glyceraldehyde-3-phosphate dehydrogenase (GAPDH) were obtained from Cell Signaling Technology (Danvers, MA, USA).

### 2.2. Experimental Models

Male C57BL/6 mice (8-10 weeks old) received a single intratracheal injection of LPS (5 mg/kg) for 12 h to induce ALI in vivo, while an equal volume of saline was used as the negative control. In a separate study, the mice were intratracheally injected with a lethal dose of LPS (25 mg/kg) for survival analysis [3, 14]. For the treatment of *miR-31-5p* duplexes, the mice were intravenously treated with *miR-31-5p* agomir, antagomir, and their negative controls at indicated doses for 3 consecutive days before LPS injection according to a previous study [14]. To inhibit AMPK*α* in mice, CpC (20 mg/kg) was intraperitoneally injected every two days from 1 week before *miR-31-5p* manipulation [20]. For Cab39 knockdown in lung tissue, the mice were exposed to a single intravenous injection of 2 × 10^7^ PFU sh*Cab39* or sh*Ctrl* as a control [21]. All experimental procedures were in accordance with the *Animal Research: Reporting of In Vivo Experiments* (ARRIVE) guidelines and also approved by the Animal Ethics Committee of Renmin Hospital of Wuhan University (approval no. WDRM 20190114).

### 2.3. Pulmonary Function Evaluation

Pulmonary function was calculated from the continuous respiratory data using the Buxco system (Sharon, CT, USA). Airway resistance, dynamic lung compliance, and pulmonary ventilation were carried out in anesthetized mice after a brief acclimation to the chamber.

### 2.4. Blood Gas Analysis

A heparinized PE10 polyethylene catheter was used to collect blood samples from the right common carotid artery of the mice, and then the blood gas parameters were analyzed by an IL GEM® Premier 3000 blood gas analyzer.

### 2.5. Lung Wet-to-Dry Ratio Calculation

After they were excised, the lungs were blotted dry and weighed immediately to obtain the wet weight. Next, the lungs were desiccated in an oven at 80°C for 4 days until constant weight to get the dry weight [3]. The lung wet-to-dry ratio was calculated to assess pulmonary edema.

### 2.6. Bronchoalveolar Lavage Fluid (BALF) Measurement

After euthanasia, the mice received intratracheal injections of 1 mL ice-cold phosphate-buffered saline (PBS, pH = 7.4) for 3 times to collect the BALF samples, which were then centrifuged at 1500 rpm for 5 min at 4°C to pellet the cells [3, 4]. The cell-free supernatants were stored at -80°C for detecting cytokines and total proteins. The sedimented cell pellets were resuspended in 0.5 mL PBS and counted by Wright-Giemsa's staining and a hemocytometer. Total protein concentrations were directly measured using a BCA protein assay kit, while the inflammatory cytokines were measured using the enzyme-linked immunosorbent assay (ELISA) kits.

### 2.7. Cell Culture and Treatments

MH-S, a murine alveolar macrophage cell line, was purchased from ATCC (Manassas, VA) and cultured in RPMI 1640 medium containing 10% fetal bovine serum. Cells were preincubated with *miR-31-5p* agomir (50 nmol/L), antagomir (100 nmol/L), or their negative controls for 24 h using the Lipofectamine 6000 Reagent (Beyotime; Shanghai, China), followed by LPS stimulation (100 ng/mL) for an additional 6 h as previously described [14]. For AMPK*α* inhibition, the cells were pretreated with CpC (20 *μ*mol/L) at 12 h before *miR-31-5p* manipulation [20]. For Cab39 silence, the macrophages were preinfected with lentivirus carrying sh*Cab39* (MOI = 50) or sh*Ctrl* for 4 h and then maintained for an additional 24 h before *miR-31-5p* manipulation.

### 2.8. Western Blot and Quantitative Real-Time PCR

Total protein samples from cultured cells or lung tissues were homogenized with RIPA lysis buffer, and then the protein concentrations were measured using the BCA protein assay kit [22–24]. Next, equal amounts of proteins were separated by SDS-PAGE gel and electrotransferred onto polyvinylidene fluoride membranes. The membranes were then blocked in 5% nonfat milk at room temperature for 1.5 h, followed by incubation with primary antibodies at 4°C overnight. On the second day, the protein bands were probed by peroxidase-conjugated secondary antibodies at room temperature for 1 h and then scanned using the Image Lab Analysis Software (Bio-Rad; Hercules, CA, USA) under visualization by an electrochemiluminescence reagent. Total RNA was extracted using the TRIzol Reagent and then reversely transcribed to cDNA by a reverse transcriptase kit (Takara; Tokyo, Japan) [25–28]. Real-time PCR was performed using the SYBR Green Master Mix (Takara Bio Inc.) on a CFX96 Touch™ Detection System (Bio-Rad; Hercules, CA, USA). The expression of mRNA was normalized to *Gapdh*, while the *miR-31-5p* level was corrected on *U6*. The primer sequences were as follows: *miR-31-5p*—forward, 5′-CGAGGCAAGATGCTGGCA-3′ and reverse, 5′-AGTGCAGGGTCCGAGGTATT-3′; *U6*—forward, 5′-GCTTCGGCAGCACATATACTAA-3′ and reverse, 5′-AACGCTTCACGAATTTGCGT-3′; *Il-6*—forward, 5′-AGTTGCCTTCTTGGGACTGA-3′ and reverse, 5′-TCCACGATTTCCCAGAGAAC-3′; *Tnf-α*—forward, 5′-CCCTCACACTCAGATCATCTTCT-3′ and reverse, 5′-GCTACGACGTGGGCTACAG-3′; *Cab39*—forward, 5′-CCGTTCCCATTTGGCAAGTCT-3′ and reverse, 5′-ACAGAATTTCTTTCATGGCGACC-3′; *Gapdh*—forward, 5′-AGGTCGGTGTGAACGGATTTG-3′ and reverse, 5′-TGTAGACCATGTAGTTGAGGTCA-3′.

### 2.9. Cytokine Detection

The concentrations of IL-6, TNF-*α*, IL-1*β*, and IL-18 in BALF, lung tissue, or cell medium were measured using the available kits according to the manufacturer's instructions. The data were calculated by comparing the optical density with the standard curve.

### 2.10. Enzymatic Activity Measurement

The activities of LDH, MPO, caspase-1, SOD, CAT, and Gpx in lung tissue or macrophages were determined by the commercial kits following the manufacturer's instructions.

### 2.11. Detection of Intracellular ROS and Oxidative Products

The lung homogenates or cell lysates were incubated with DCFH-DA (50 *μ*mol/L) at 37°C for 30 min in the dark [29, 30]. DCF fluorescence intensities were then measured using a Bio-Tek microplate reader at the excitation and emission wavelengths of 485 nm and 535 nm, respectively. The levels of oxidative products for lipids (MDA and 4-HNE) or proteins (3-NT) were detected using the commercial kits according to the manufacturer's instructions.

### 2.12. Dual-Luciferase Reporter Gene Assay

The wild type or mutant 3′-UTR of Cab39 was cloned into the pGL3 plasmid (Promega; Madison, WI, USA) containing a luciferase report gene, which was then cotransfected with the *miR-31-5p* agomir or AgNC using the Lipofectamine 6000 Reagent (Beyotime; Shanghai, China). The cells were cultured for 48 h and then collected to detect the firefly and Renilla luciferase activities by a Dual-Luciferase Reporter Assay System (Promega; Madison, WI, USA) [31–33].

### 2.13. Statistical Analysis

All data were presented as the mean ± standard deviation (SD) and analyzed using the SPSS 22.0 software. An unpaired two-tailed Student *t*-test was performed to compare the significance between two groups, whereas the differences among three or more groups were calculated by one-way ANOVA analysis followed by Tukey's post hoc test. Statistical significance was defined as *P* < 0.05.

## 3. Results

### 3.1. *miR-31-5p* Antagomir Alleviates LPS-Induced ALI in Mice

**Figure 1:**
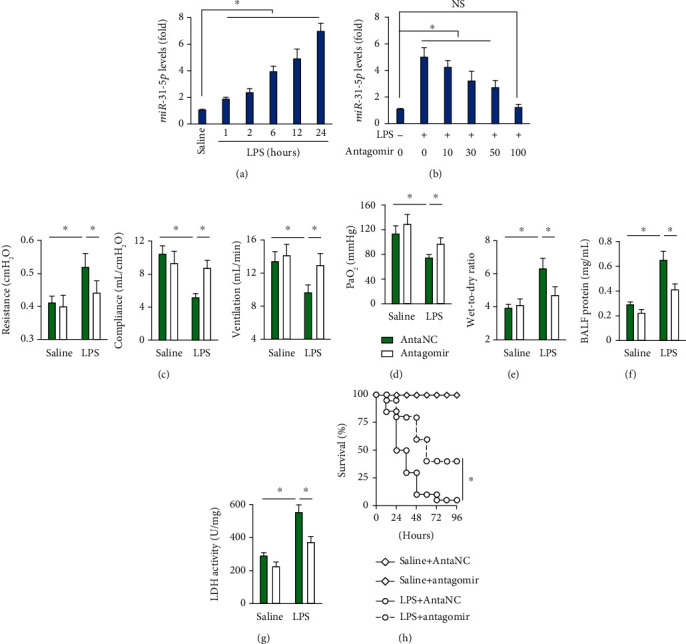
*miR-31-5p* antagomir alleviates LPS-induced ALI in mice. (a) Pulmonary *miR-31-5p* levels after LPS (5 mg/kg) treatment for the indicated times (*n* = 6). (b) Pulmonary *miR-31-5p* levels in mice with different doses of *miR-31-5p* antagomir treatment upon LPS (5 mg/kg) stimulation for 12 h (*n* = 6). (c) The mice were pretreated with *miR-31-5p* antagomir (100 mg/kg) for 3 consecutive days and then received LPS (5 mg/kg) stimulation for an additional 12 h. Respiratory function, including airway resistance, lung compliance, and pulmonary ventilation was detected in mice (*n* = 6). (d) PaO_2_ in mice (*n* = 6). (e) Lung wet-to-dry ratio (*n* = 8). (f) Total protein levels in BALF (*n* = 6). (g) LDH activities in lung tissue (*n* = 6). (h) The survival rate in mice with or without *miR-31-5p* antagomir protection after a lethal dose (25 mg/kg) of LPS injection (*n* = 20). Data are mean ± SD. ^∗^*P* < 0.05 versus the matched group. NS indicates no significance.

We first explored whether *miR-31-5p* expression was altered during ALI, and the data identified an upregulation of *miR-31-5p* levels in lung tissue upon LPS injection (Figure 1(a)). As shown in Figure 1(b), *miR-31-5p* antagomir normalized the aberrant *miR-31-5p* expression in LPS-injured lungs to a physiological level at the dose of 100 mg/kg; therefore, we selected this dose of *miR-31-5p* antagomir in our further study. Intriguingly, *miR-31-5p* antagomir treatment decreased airway resistance and increased lung compliance and pulmonary ventilation in mice with LPS-induced ALI (Figure 1(c)). Accordingly, the reduced partial pressure of arterial oxygen (PaO_2_) was also prevented by *miR-31-5p* antagomir (Figure 1(d)). LPS injection caused severe pulmonary edema and protein leakage, which were attenuated in mice with *miR-31-5p* antagomir treatment, as confirmed by the decreased wet-to-dry ratio of lung tissue and BALF protein concentrations (Figures 1(e) and 1(f)). LDH is a critical marker of cellular damage, and the increased LDH activity in lung tissue after LPS injection was remarkably decreased by *miR-31-5p* antagomir (Figure 1(g)). Moreover, *miR-31-5p* antagomir treatment could improve the survival status of LPS-challenged mice (Figure 1(h)). Collectively, we conclude that *miR-31-5p* antagomir alleviates lung injury and enhances the respiratory function of LPS-treated ALI mice.

### 3.2. *miR-31-5p* Agomir Aggravates LPS-Induced ALI in Mice

**Figure 2:**
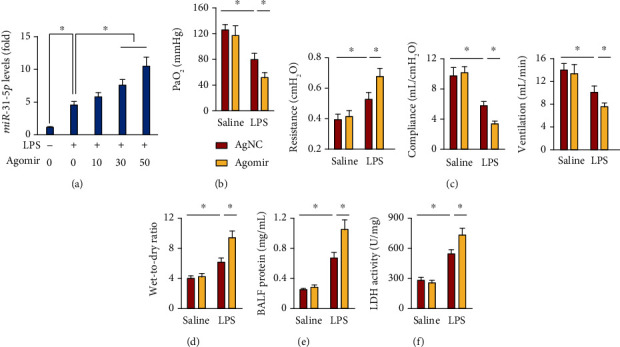
*miR-31-5p* agomir aggravates LPS-induced ALI in mice. (a) Pulmonary *miR-31-5p* levels in mice with different doses of *miR-31-5p* agomir treatment upon LPS (5 mg/kg) stimulation for 12 h (*n* = 6). (b) The mice were pretreated with *miR-31-5p* agomir (50 mg/kg) for 3 consecutive days and then received LPS (5 mg/kg) stimulation for an additional 12 h. PaO_2_ was detected in mice (*n* = 6). (c) Respiratory function, including airway resistance, lung compliance, and pulmonary ventilation in mice (*n* = 6). (d) Lung wet-to-dry ratio (*n* = 8). (e) Total protein levels in BALF (*n* = 6). (f) LDH activity in lung tissue (*n* = 6). Data are mean ± SD. ^∗^*P* < 0.05 versus the matched group.

We then investigated whether the increased *miR-31-5p* expression could accelerate LPS-induced ALI in mice. As depicted in Figure 2(a), *miR-31-5p* agomir elicited almost two times expression of *miR-31-5p* in lung tissue upon LPS stimulation at the dose of 50 mg/kg; thus, we used this dose in the next study. As mentioned above, the mice with LPS injection had lower PaO_2_ that was further decreased by *miR-31-5p* agomir treatment (Figure 2(b)). Besides, the airway resistance was further decreased, and the lung compliance and pulmonary ventilation were further decreased in *miR-31-5p* agomir-treated mice upon LPS stimulation (Figure 2(c)). LPS-induced pulmonary edema and injury were also exacerbated in mice with *miR-31-5p* agomir treatment (Figures 2(d)–2(f)). In the survival study, the mice treated with *miR-31-5p* agomir all died at 36 h post-LPS injection (data not shown). These findings indicate that *miR-31-5p* agomir aggravates LPS-induced ALI in mice.

### 3.3. *miR-31-5p* Antagomir Reduces Intrapulmonary Inflammation in LPS-Treated Mice

**Figure 3:**
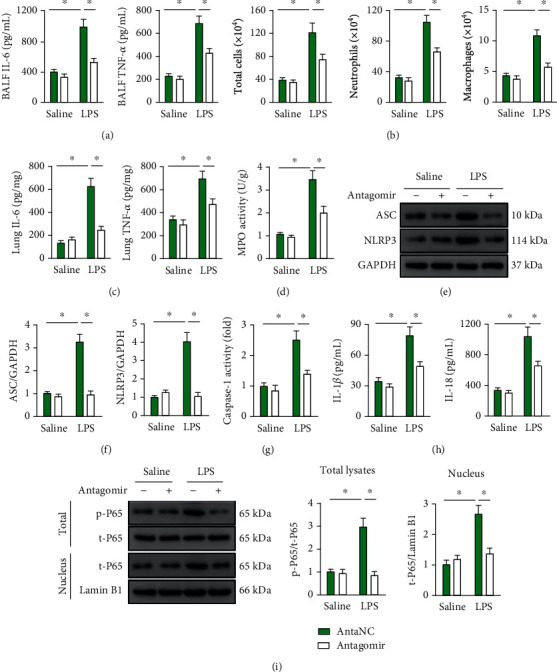
*miR-31-5p* antagomir reduces intrapulmonary inflammation in LPS-treated mice. (a and b) The mice were pretreated with *miR-31-5p* antagomir (100 mg/kg) for 3 consecutive days and then received LPS (5 mg/kg) stimulation for an additional 12 h. IL-6, TNF-*α*, and the number of inflammatory cells were measured in BALF (*n* = 6). (c and d) The levels of IL-6, TNF-*α*, and MPO activities in lung tissue (*n* = 6). (e and f) Representative western blot images and the statistical data (*n* = 6). (g) Caspase-1 activities in lung tissue (*n* = 6). (h) The levels of IL-1*β* and IL-18 in lung tissue (*n* = 6). (i) Representative western blot images and the statistical data (*n* = 6). Data are mean ± SD. ^∗^*P* < 0.05 versus the matched group.

We next evaluated the effect of *miR-31-5p* antagomir on LPS-induced intrapulmonary inflammation in mice and found that *miR-31-5p* antagomir effectively decreased the levels of BALF IL-6 and TNF-*α* (Figure 3(a)). Besides, *miR-31-5p* antagomir pretreatment remarkably reduced the accumulation of total cells, macrophages, and neutrophils in BALF (Figure 3(b)). Accordingly, the levels of IL-6 and TNF-*α*, as well as MPO activity in the lungs of ALI mice, were also suppressed by *miR-31-5p* antagomir treatment (Figures 3(c) and 3(d)). NLRP3 inflammasome plays pivotal roles in inflammatory response and LPS-induced ALI [3, 4]. Here, we found that *miR-31-5p* antagomir decreased NLRP3 inflammasome component expression, including ASC and NLRP3, and also strongly reduced caspase-1 activity in LPS-treated lungs (Figures 3(f) and 3(g)). As expected, the releases of IL-1*β* and IL-18 were also suppressed (Figure 3(h)). NF-*κ*B acts as an inflammation-related transcription factor that is required for NLRP3 inflammasome activation and ALI progression [34]. Interestingly, NF-*κ*B phosphorylation and nuclear accumulation were markedly suppressed in *miR-31-5p* antagomir-treated lungs upon LPS injection (Figure 3(i)). Taken together, we prove that *miR-31-5p* antagomir reduces intrapulmonary inflammation in LPS-treated mice.

### 3.4. *miR-31-5p* Antagomir Mitigates Intrapulmonary Oxidative Damage in LPS-Treated Mice

**Figure 4:**
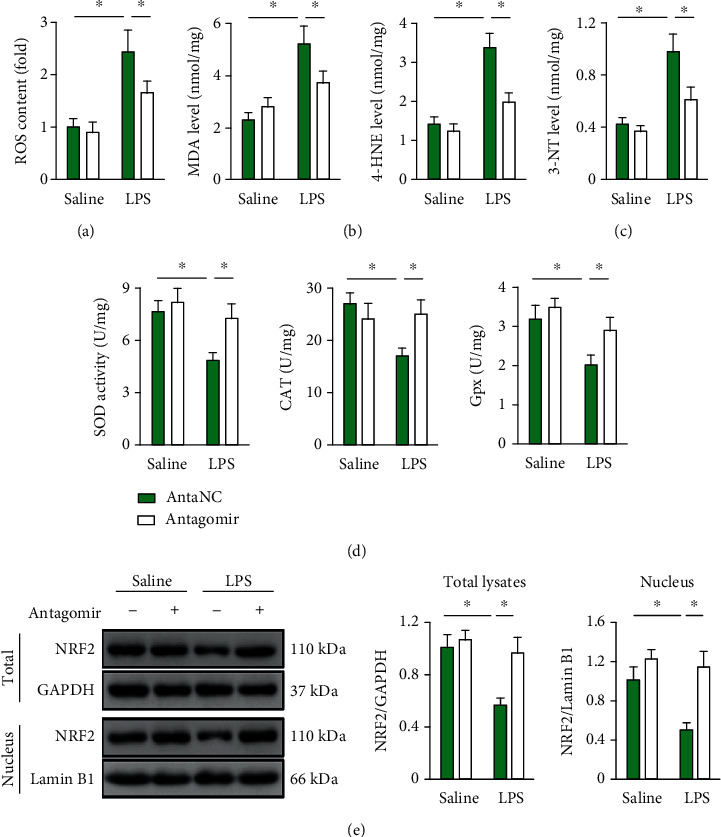
*miR-31-5p* antagomir mitigates intrapulmonary oxidative damage in LPS-treated mice. (a) The mice were pretreated with *miR-31-5p* antagomir (100 mg/kg) for 3 consecutive days and then received LPS (5 mg/kg) stimulation for an additional 12 h. ROS content was measured by DCFH-DA in lung tissue (*n* = 6). (b and c) The levels of MDA, 4-HNE, and 3-NT were detected in lung tissue (*n* = 6). (d) Total SOD activities, CAT activities, and Gpx activities in lung tissue (*n* = 6). (e) Representative western blot images and the statistical data (*n* = 6). Data are mean ± SD. ^∗^*P* < 0.05 versus the matched group.

Oxidative stress is the other feature of LPS-induced ALI [3, 14]. As shown in Figures 4(a)–4(c), the lungs with LPS stimulation had increased ROS generation and oxidative products for lipids (MDA and 4-HEN) and proteins (3-NT) that were significantly decreased by *miR-31-5p* antagomir. SOD, CAT, and Gpx are three key intracellular antioxidant enzymes to scavenge the excessive free radicals for redox homeostasis. We observed that *miR-31-5p* antagomir restored total SOD, CAT, and Gpx activities in lungs with LPS injury (Figure 4(d)). NRF2 is an important transcription factor in regulating the expression of numerous antioxidant enzymes, and we thus detected whether *miR-31-5p* antagomir affects the NRF2 pathway. As depicted in Figure 4(e), LPS significantly decreased NRF2 expression and nuclear accumulation in lung tissue, yet to a lesser extent compared to those in lung tissue with *miR-31-5p* antagomir protection. Altogether, these data demonstrate an antioxidant role of *miR-31-5p* antagomir in LPS-induced ALI.

### 3.5. *miR-31-5p* Agomir Exacerbates LPS-Induced Intrapulmonary Inflammation and Oxidative Damage in Mice

In contrast, the mice treated with *miR-31-5p* agomir had increased BALF IL-6 and TNF-*α* levels, and LPS-triggered infiltrations of inflammatory cells to the lung tissue were also augmented by *miR-31-5p* agomir (S1A and S1B). Correspondingly, pulmonary IL-6 and TNF-*α* levels and MPO activity were increased in *miR-31-5p* agomir-treated mice upon LPS stimulation (Figures S1C and S1D). Besides, LPS-induced activation of NLRP3 inflammasome was further promoted in *miR-31-5p*-overexpressed lungs, as verified by the elevated IL-1*β*, IL-18, and caspase-1 activities (Figures S1E and S1F). Meanwhile, *miR-31-5p* agomir evidently increased ROS generation in LPS-injured lungs, followed by massive production of MDA, 4-HNE, and 3-NT (Figures S1G-S1I). Overall, the above findings identify a necessary role of *miR-31-5p* in LPS-induced ALI in mice; however, the expression pattern of *miR-31-5p*, either by overexpression or inhibition, did not affect inflammation, oxidative stress, or pulmonary function under basal conditions.

### 3.6. *miR-31-5p* Modulates Inflammation and Oxidative Stress in LPS-Treated Macrophages

**Figure 5:**
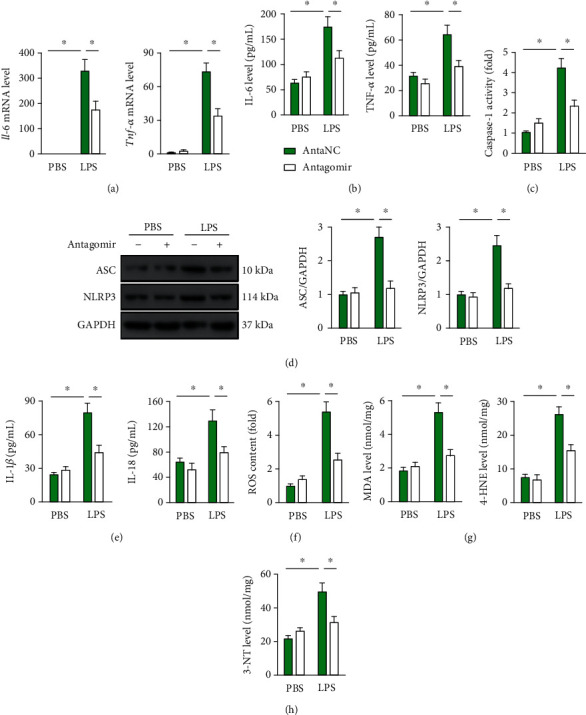
*miR-31-5p* antagomir decreases inflammation and oxidative stress in LPS-treated macrophages. (a) MH-S alveolar macrophages were preincubated with *miR-31-5p* antagomir (100 nmol/L) or the negative control for 24 h and then received LPS stimulation (100 ng/mL) for an additional 6 h. Relative mRNA levels of *Il-6* and *Tnf-α* in the macrophages (*n* = 6). (b) IL-6 and TNF-*α* levels in the medium (*n* = 6). (c) Cellular caspase-1 activity (*n* = 6). (d) Representative western blot images and the statistical data (*n* = 6). (e) IL-1*β* and IL-18 levels in the medium (*n* = 6). (f) Intracellular ROS content was measured by DCFH-DA in the macrophages (*n* = 6). (g and h) The levels of MDA, 4-HNE, and 3-NT were detected in the macrophages (*n* = 6). Data are mean ± SD. ^∗^*P* < 0.05 versus the matched group.

Based on the in vivo data, we next investigated whether *miR-31-5p* could modulate LPS-induced inflammation and oxidative stress in MH-S alveolar macrophages in vitro. Consistent with the in vivo findings, macrophages with LPS treatment had increased expression and secretion of IL-6 and TNF-*α* that were suppressed by *miR-31-5p* antagomir incubation (Figures 5(a) and 5(b)). *miR-31-5p* antagomir also suppressed LPS-induced activation of NLRP3 inflammasome in MH-S cells, as confirmed by the decreased caspase-1 activity, ASC/NLRP3 protein levels, and IL-1*β* and IL-18 releases (Figures 5(c)–5(e)). In addition, we observed that *miR-31-5p* antagomir suppressed intracellular ROS production and decreased MDA, 4-HNE, and 3-NT formation in macrophages upon LPS stimulation (Figures 5(f)–5(h)). Conversely, the macrophages with *miR-31-5p* agomir treatment had increased inflammatory response and oxidative damage upon LPS stimulation (Figures S2A–S2E). These findings imply that *miR-31-5p* modulates inflammation and oxidative stress in LPS-treated macrophages.

### 3.7. *miR-31-5p* Antagomir Prevents LPS-Induced Inflammation, Oxidative Stress, and ALI via Activating AMPK*α* In Vivo and In Vitro

**Figure 6:**
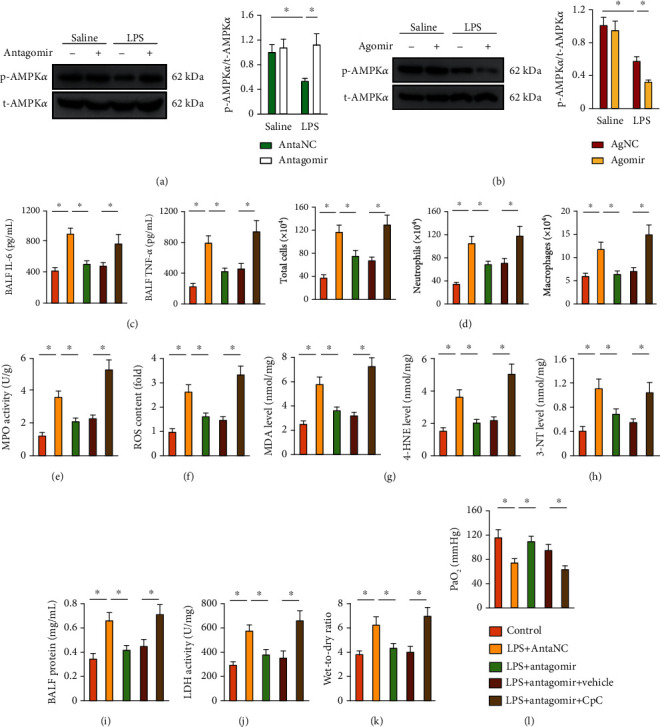
*miR-31-5p* antagomir prevents LPS-induced inflammation, oxidative stress, and ALI via activating AMPK*α* in vivo. (a and b) The mice were pretreated with *miR-31-5p* agomir (50 mg/kg) or antagomir (100 mg/kg) for 3 consecutive days and then received LPS (5 mg/kg) stimulation for an additional 12 h. Representative western blot images and the statistical data (*n* = 6). (c and d) CpC (20 mg/kg) was intraperitoneally injected every two days from 1 week before *miR-31-5p* manipulation to inhibit AMPK*α* in mice. IL-6, TNF-*α*, and the number of inflammatory cells were measured in BALF (*n* = 6). (e) MPO activities in lung tissue (*n* = 6). (f) ROS content was measured by DCFH-DA in lung tissue (*n* = 6). (g and h) The levels of MDA, 4-HNE, and 3-NT were detected in lung tissue (*n* = 6). (i) Total protein levels in BALF (*n* = 6). (j) LDH activity in lung tissue (*n* = 6). (k) Lung wet-to-dry ratio (*n* = 8). (l) PaO_2_ was detected in mice (*n* = 6). Data are mean ± SD. ^∗^*P* < 0.05 versus the matched group.

We next tried to clarify whether AMPK*α* was required for the protective effects of *miR-31-5p* antagomir against LPS-induced ALI. As depicted in Figure 6(a), LPS-induced AMPK*α* inactivation was prevented by *miR-31-5p* antagomir, while it was further exacerbated by *miR-31-5p* agomir (Figures 6(a) and 6(b)). To verify the involvement of AMPK*α*, the mice were injected with CpC to inhibit AMPK*α* activity in *miR-31-5p* antagomir-treated mice. As depicted in Figures 6(c)–6(e), CpC abrogated the inhibitory effects of *miR-31-5p* antagomir against LPS-induced intrapulmonary inflammation and leukocyte infiltration. Besides, *miR-31-5p* antagomir lost its antioxidant capacity in CpC-treated lungs (Figures 6(f)–6(h)). As mentioned above, *miR-31-5p* antagomir protected against LPS-induced pulmonary injury, edema, and dysfunction, yet it failed to do so in the lungs with AMPK*α* inhibition (Figures 6(i)–6(l)). To further determine the necessity of AMPK*α* in *miR-31-5p* antagomir-mediated anti-inflammatory and antioxidant effects in vitro, the MH-S alveolar macrophages were pretreated with CpC. As shown in Figures S3A and S3B, AMPK*α* inhibition notably blunted the anti-inflammatory effect of *miR-31-5p* antagomir in vitro, as evidenced by the unaffected IL-6, TNF-*α*, IL-1*β*, and IL-18 levels. Of note, *miR-31-5p* antagomir decreased the levels of ROS content, MDA, 4-HNE, and 3-NT formation in macrophages, but not in those with CpC treatment (Figures S3C–S3E). These studies define AMPK*α* as a potential molecular target for the protective effects of *miR-31-5p* antagomir against LPS-induced ALI.

### 3.8. *miR-31-5p* Antagomir Activates AMPK*α* via Increasing Cab39 Expression

**Figure 7:**
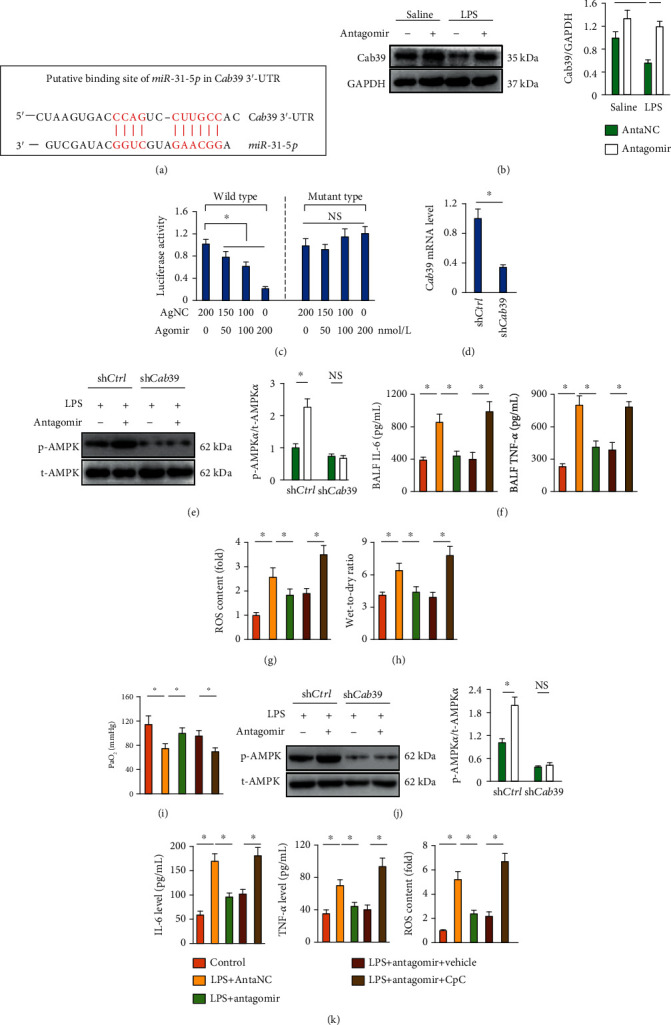
*miR-31-5p* antagomir activates AMPK*α* via increasing Cab39 expression. (a) Putative binding site of *miR-31-5p* in *Cab39* 3′-UTR. (b) Representative western blot images and the statistical data (*n* = 6). (c) Luciferase activity in cells transfected with wild type of mutant *Cab39* 3′-UTR after *miR-31-5p* agomir treatment (*n* = 6). (d) *Cab39* mRNA level in lung tissue (*n* = 6). (e) Representative western blot images and the statistical data (*n* = 6). (f) IL-6 and TNF-*α* levels were measured in BALF (*n* = 6). (g) ROS content was measured by DCFH-DA in lung tissue (*n* = 6). (h) Lung wet-to-dry ratio (*n* = 8). (i) PaO_2_ was detected in mice (*n* = 6). (j) Representative western blot images and the statistical data (*n* = 6). (k) IL-6 and TNF-*α* levels in the medium and intracellular ROS content in the macrophages (*n* = 6). Data are mean ± SD. ^∗^*P* < 0.05 versus the matched group. NS indicates no significance.

We finally investigated the possible pathway through which *miR-31-5p* antagomir activated AMPK*α*. TargetScan software was used to predict the potential target of *miR-31-5p*, and we observed a putative binding site of *miR-31-5p* in the 3′-UTR of *Cab39* that serves as a scaffold protein for AMPK*α* activation (Figure 7(a)) [35]. Besides, LPS-elicited Cab39 suppression in lungs was preserved by *miR-31-5p* antagomir (Figure 7(b)). To examine whether *miR-31-5p* can directly bind to the 3′-UTR of Cab39, a dual-luciferase reporter gene assay was performed. As shown in Figure 7(c), luciferase activities were significantly inhibited when *miR-31-5p* was cotransfected with the luciferase plasmid harboring wild type *Cab39* 3′-UTR, yet it failed to do the same when the binding site was mutated. To strengthen the role of Cab39 in AMPK*α* activation by *miR-31-5p* antagomir, we knocked down Cab39 expression in lung tissue using lentiviral vectors (Figure 7(d)). As expected, Cab39 silence blocked AMPK*α* activation in *miR-31-5p* antagomir-treated murine lungs, accompanied by increased levels of BALF IL-6 and TNF-*α* as well as ROS generation (Figures 7(e)–7(g)). Accordingly, the beneficial effects of *miR-31-5p* antagomir against LPS-induced pulmonary edema and dysfunction were also retarded after Cab39 silence (Figures 7(h) and 7(i)). Consistent with the in vivo data, AMPK*α* activation by *miR-31-5p* antagomir was counteracted in macrophages with sh*Cab39* infection, and the inhibitory effects on inflammation and ROS generation were also abolished (Figures 7(j) and 7(k)). Therefore, we summarize that Cab39 is required for *miR-31-5p* antagomir-mediated AMPK*α* activation and the subsequent pulmonary protection against LPS-induced ALI.

## 4. Discussion

Our present study indicates that *miR-31-5p* is upregulated in murine lungs upon LPS stimulation and that this upregulation is instrumental for the provocation of inflammation and oxidative damage both in mice and in cultured macrophages. *miR-31-5p* antagomir attenuates, while *miR-31-5p* agomir exacerbates pulmonary injury and dysfunction in LPS-treated mice. Besides, we report that *miR-31-5p* antagomir reduces proinflammatory cytokine secretion, ROS generation, and lung injury via activating AMPK*α*, and conversely, AMPK*α* inhibition by CpC blocks the pulmonary protection in vivo and in vitro. Further data identify Cab39 as a direct target of *miR-31-5p*, and *miR-31-5p* antagomir prevents LPS-induced Cab39 downregulation and thus activates AMPK*α*. We report here for the first time that endogenous *miR-31-5p* is a key pathogenic factor for inflammation and oxidative damage during LPS-induced ALI.

Excessive inflammation and oxidative stress are thought to play critical roles in the initiation and progression of ALI [3, 36]. Inflammatory cells and the proinflammatory cytokine notably trigger free radical overproduction, and ROS in turn activates the inflammatory programs, which create a vicious cycle to accelerate inflammation and oxidative damage and provoke the occurrence of ALI [5]. Upon LPS stimulation, NF-*κ*B is phosphorylated and translocated from the cytoplasm to the nucleus, where it binds to its consensus sequence on the promoter-enhancer region of targeted genes and drives the transcription of inflammatory cytokines [37]. Besides, the local inflammation also recruits circulating leukocytes into the lung tissue and further amplifies the inflammatory response. Inflammasomes function as the molecular scaffolds for inflammatory response and are essential for the maturation of multiple procytokines [38–40]. Emerging studies demonstrate a central role of NLRP3 inflammasome in the pathological process of ALI. Upon stimulation, the ASC adaptor interacts with the NLRP3 scaffold to activate caspase-1, which then proteolytically cleaves the precursors of multiple proinflammatory cytokines and releases the mature forms, including IL-1*β* and IL-18 [3, 4, 14]. Consistently, LPS stimulation activated NF-*κ*B and NLRP3 inflammasome in the present study, and elicited increases of multiple proinflammatory cytokines in lung tissues and cultured alveolar macrophages, which were notably blunted by *miR-31-5p* antagomir treatment. Oxidative stress is also implicated in the development of ALI. Free radicals, such as hydrogen peroxide and superoxide anion, are increased in lung tissue in response to LPS injury, which directly cause lipid and protein peroxidation, resulting in the injury and death of lung cells [3]. Besides, ROS overproduction by LPS challenge in lung tissue also triggers the dissociation of thioredoxin interacting protein (TXNIP) from thioredoxin, which then binds to and activates NLRP3 inflammasome [39, 41]. The redox sensor NRF2 is physiologically retained in the cytoplasm by Kelch-like ECH-associated protein 1 (KEAP1), but it detaches from KEAP1 upon oxidative stress and subsequently translocates to the nucleus to trigger the antioxidant response [22]. Herein, we observed that *miR-31-5p* antagomir restored NRF2 expression and nuclear accumulation in LPS-induced ALI, thereby preventing ROS overproduction and oxidative damage.

*miR-31-5p* is a well-known tumor-associated microRNA and participates in the progression of various tumors, including lung cancer, colorectal cancer, and hepatocellular carcinoma [16, 17]. Emerging studies indicate that *miR-31-5p* also plays indispensable roles in maintaining the pathophysiological homeostasis in noncancerous tissues. Liu et al. proved that *miR-31-5p* could repress the proliferation and differentiation of tongue myoblasts [42]. Results from Ji et al. indicated that *miR-31-5p* silence significantly alleviated the doxorubicin-induced myocardial apoptosis and cardiac dysfunction in mice [43]. A very recent study by Toyonaga et al. demonstrated that *miR-31-5p* was required for colonic epithelial cell integrity and could predict the clinical outcomes in patients with Crohn's disease [19]. We herein for the first time identified the pathogenic role of *miR-31-5p* in LPS-induced inflammation, oxidative stress, and ALI. Cab39 functions as a scaffold protein of liver kinase B1 (LKB1), an upstream kinase of AMPK*α*, and stabilizes the LKB1's activity through forming a heterotrimeric complex with the STE20-related kinase adaptor in the cytoplasm [35, 44]. In the current study, we observed that *miR-31-5p* directly bound to the 3′-UTR of Cab39 to inhibit its expression, while *miR-31-5p* antagomir restored the Cab39 protein level, accompanied by an increased AMPK*α* phosphorylation and the improvement on LPS-induced inflammation, oxidative stress, and pulmonary dysfunction. Despite being famous as an energy sensor, AMPK*α* also plays indispensable roles in regulating inflammation and oxidative stress. Its activation was reported to reduce ROS generation and prevent diabetes-, sepsis-, or doxorubicin-induced cardiac injury [45–47]. In line with our findings, some investigators proved that AMPK*α* notably enhanced the expression and nuclear translocation of NRF2 to scavenge the excessive free radicals [3, 10]. Besides, a recent study found that AMPK*α* activation effectively decreased p47phox expression and phosphorylation, thereby decreasing the generation of free radicals [48]. Previous data indicated that the inhibition of oxidative stress by AMPK*α* significantly blocked NLRP3 inflammasome activation and inflammatory damage [3, 10, 48]. NF-*κ*B is essential for NLRP3 inflammasome activation, and the findings from us and other laboratories demonstrated that NF-*κ*B inhibition was sufficient to alleviate the activation of NLRP3 inflammasome [34]. Moreover, results from Chen et al. revealed that AMPK*α* regulated dynamin-related protein 1-mediated mitochondrial fission and thereby restrained NLRP3 inflammasome activation [49]. Collectively, our data defined *miR-31-5p* as a promising therapeutic target for the treatment of ALI.

Of note, there exist some limitations in the current study. First, an invasive respiratory mechanics as previously described would be more appropriate to evaluate pulmonary function [50]. Second, whether *miR-31-5p* affects the production of free radicals need to be confirmed. Besides, the role of *miR-31-5p* in other pulmonary cells, e.g., the lung epithelial cells, during the progression of ALI should be investigated in further studies.

## Data Availability

The data that support the findings of this study are available from the corresponding authors upon reasonable request.

## Supplementary Materials

Supplementary Materials Figure S1: *miR-31-5p* agomir exacerbates LPS-induced intrapulmonary inflammation and oxidative damage in mice. Figure S2: *miR-31-5p* agomir increases inflammation and oxidative stress in LPS-treated macrophages. Figure S3: AMPK*α* inhibition abolishes the anti-inflammatory and antioxidant effects of *miR-31-5p* antagomir in LPS-treated macrophages.
