# Supplementary material for: MicroRNA-31-5p Exacerbates Lipopolysaccharide-Induced Acute Lung Injury via Inactivating Cab39/AMPKα Pathway
Source: Oxid Med Cell Longev. 2020 Oct 8;2020:8822361. doi: 10.1155/2020/8822361 (PMC7568166; doi:10.1155/2020/8822361)
Supplement: Supplementary Materials — Figure S1: miR-31-5p agomir exacerbates LPS-induced intrapulmonary inflammation and oxidative damage in mice. Figure S2: miR-31-5p agomir increases inflammation and oxidative stress in LPS-treated macrophages. Figure S3: AMPKα inhibition abolishes the anti-inflammatory and antioxidant effects of miR-31-5p antagomir in LPS-treated macrophages. [file 8822361.f1.docx]

**Supplementary materials**

**MicroRNA-31-5p exacerbates lipopolysaccharide-induced acute lung injury via inactivating Cab39/AMPKα**

MicroRNA-31-5p exacerbates ALI

Wan-li Jiang^1, *^, Kao-chang Zhao^2, *^, Wen Yuan^3, *^, Fang Zhou^4, *^, Heng-ya Song^1^, Gao-li Liu^1^, Jie Huang^1^, Jin-jing Zou^2, #^, Bo Zhao^4, #^, Song-ping Xie^1, #^

^1^ Department of Thoracic Surgery, Renmin Hospital of Wuhan University, Wuhan 430060, China

^2^ Department of Pulmonary and Critical Care Medicine, Renmin Hospital of Wuhan University, Wuhan 430060, China

^3^ Department of Laboratory Medicine, Wuhan Medical and Health Center for Women and Children, Huazhong University of Science and Technology, Wuhan 430016, China

^4^ Department of Anesthesiology, Renmin Hospital of Wuhan University, Wuhan 430060, China

^*^ These authors contributed equally to this work.

^#^ Corresponding author: Dr. Song-ping Xie, Dr. Bo Zhao, Dr. Jin-jing Zou

Department of Thoracic Surgery, Renmin Hospital of Wuhan University

No.238 Jiefang Road, Wuchang District, Wuhan 430060, China

Tel: +86-13871110636, +86-18627806783

E-mail addresses:

[songping0428@126.com](mailto:songping0428@126.com) (Dr. Song-ping Xie)

[zb14526@163.com](mailto:zb14526@163.com) (Dr. Bo Zhao)

[Zoujinjingwhu@163.com](mailto:Zoujinjingwhu@163.com) (Dr. Jin-jing Zou)


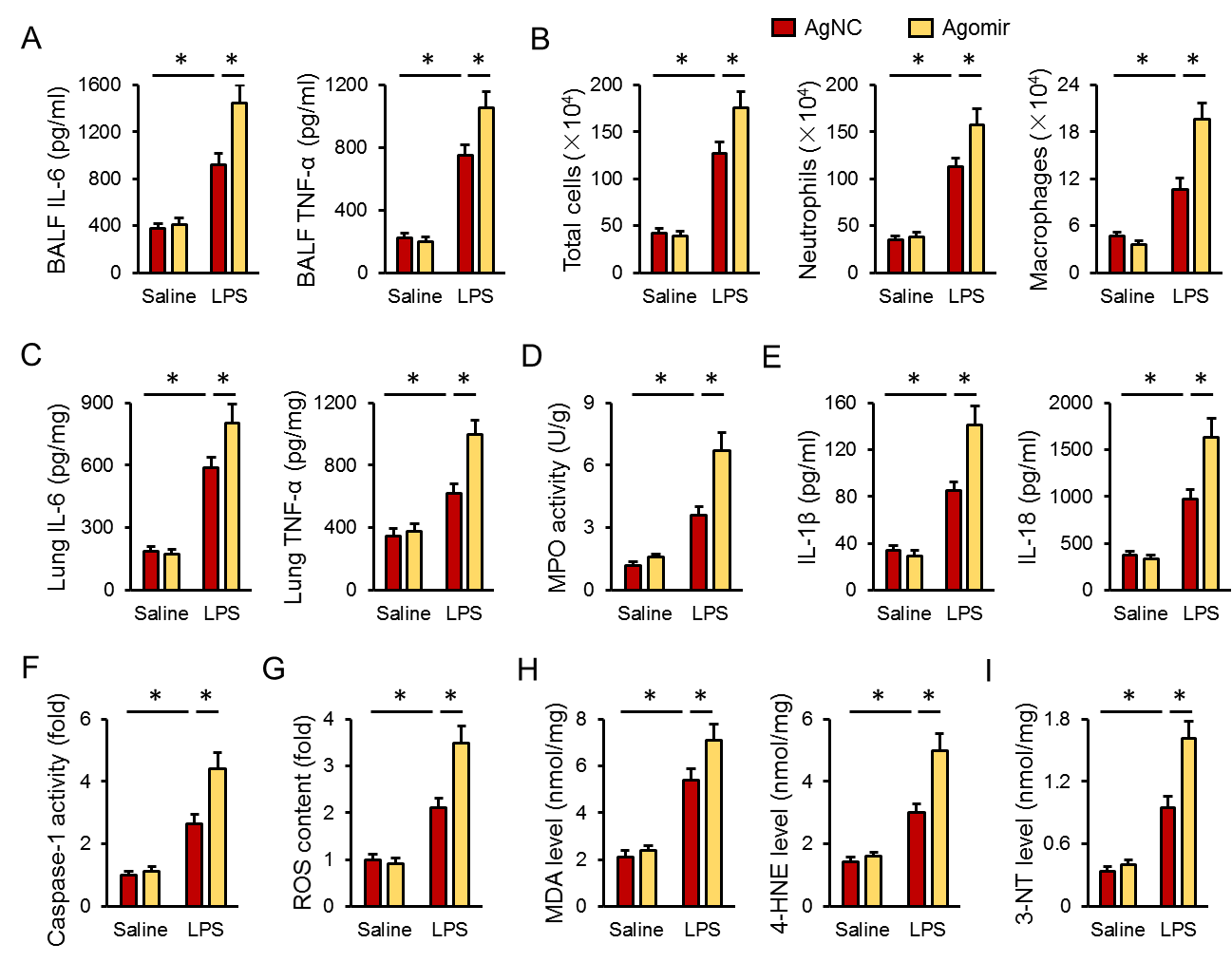


**Figure S1. *miR-31-5p* agomir exacerbates LPS-induced intrapulmonary inflammation and oxidative damage in mice. (A-B)** The mice were pretreated with *miR-31-5p* agomir (50 mg/kg) for consecutive 3 days and then received LPS (5 mg/kg) stimulation for additional 12 h. IL-6, TNF-α and the number of inflammatory cells were measured in BALF (n=6). **(C-D)** The levels of IL-6, TNF-α and MPO activities in the lung tissue (n=6). **(E)** The levels of IL-1β and IL-18 in the lung tissue (n=6). **(F)** Caspase-1 activities in the lung tissue (n=6). **(G)** ROS content was measured by DCFH-DA in the lung tissue (n=6). **(H-I)** The levels of MDA, 4-HNE and 3-NT were detected in the lung tissue (n=6). Data are mean±SD. **P*<0.05 versus the matched group.


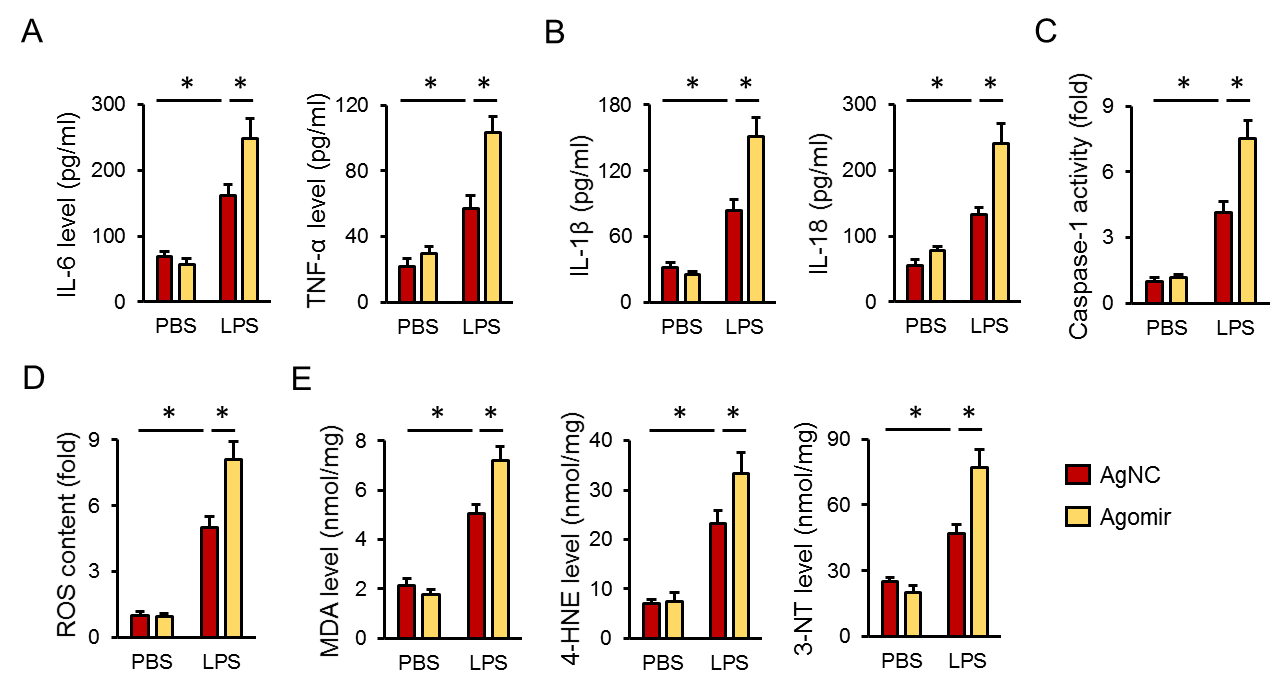


**Figure S2.** ***miR-31-5p* agomir increases inflammation and oxidative stress in LPS-treated macrophages. (A-B)** MH-S alveolar macrophages were preincubated with *miR-31-5p* agomir (50 nmol/L) or the negative control for 24 h and then received LPS stimulation (100 ng/mL) for additional 6 h. IL-6, TNF-α, IL-1β and IL-18 levels in the medium (n=6). **(C)** Cellular caspase-1 activity (n=6). **(D)** Intracellular ROS content was measured by DCFH-DA in the macrophages (n=6). **(E)** The levels of MDA, 4-HNE and 3-NT were detected in the macrophages (n=6). Data are mean±SD. **P*<0.05 versus the matched group.


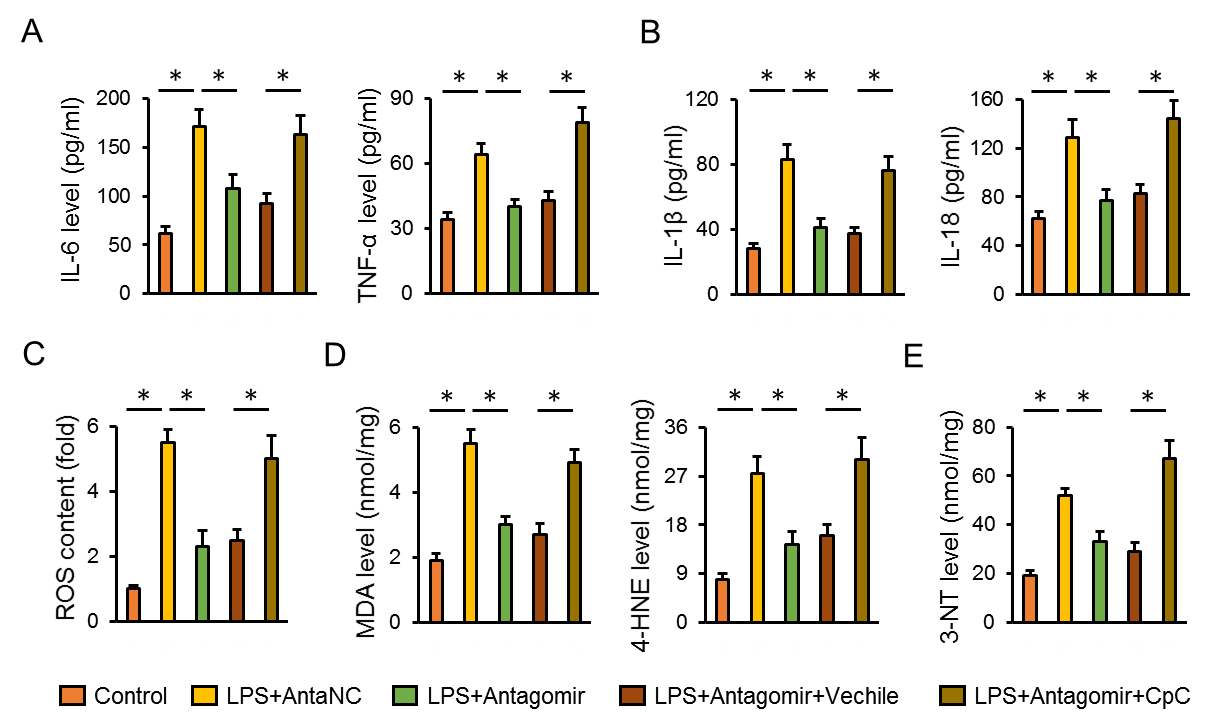


**Figure S3. AMPKα inhibition abolishes the antiinflammatory and antioxidant effects of *miR-31-5p* antagomir in LPS-treated macrophages. (A-B)** MH-S alveolar macrophages were preincubated with *miR-31-5p* antagomir (100 nmol/L) or the negative control for 24 h and then received LPS stimulation (100 ng/mL) for additional 6 h. For AMPKα inhibition, the cells were pretreated with CpC (20 μmol/L) at 12 h before *miR-31-5p* manipulation. IL-6, TNF-α, IL-1β and IL-18 levels in the medium (n=6). **(C)** Intracellular ROS content was measured by DCFH-DA in the macrophages (n=6). **(D-E)** The levels of MDA, 4-HNE and 3-NT were detected in the macrophages (n=6). Data are mean±SD. **P*<0.05 versus the matched group.
